# The effect of the UP4FUN pilot intervention on objectively measured sedentary time and physical activity in 10–12 year old children in Belgium: the ENERGY-project

**DOI:** 10.1186/1471-2458-12-805

**Published:** 2012-09-18

**Authors:** Maïte Verloigne, Elling Bere, Wendy Van Lippevelde, Lea Maes, Nanna Lien, Froydis N Vik, Johannes Brug, Greet Cardon, Ilse De Bourdeaudhuij

**Affiliations:** 1 Department of Movement and Sport Sciences, Ghent University, Ghent, Belgium; 2 Department of Public Health, Sport and Nutrition, University of Agder, Agder, Norway; 3 Department of Public Health, Ghent University, Ghent, Belgium; 4 Department of Nutrition, University of Oslo, Oslo, Norway; 5 Department of Epidemiology and Biostatistics and the EMGO Institute for Health & Care Research, VU University Medical Center, Amsterdam, The Netherlands

**Keywords:** Sedentary behaviour, Intervention, Randomized controlled trial, Children, Physical activity, Accelerometer

## Abstract

**Bakckground:**

The first aim was to examine the effect of the UP4FUN pilot intervention on children’s total sedentary time. The second aim was to investigate if the intervention had an effect on children’s physical activity (PA) level. Finally, we aimed to investigate demographic differences (i.e. age, gender, ethnicity, living status and having siblings) between children in the intervention group who improved in sedentary time and PA at post-test and children in the intervention group who worsened in sedentary time and PA at post-test.

**Methods:**

The six weeks UP4FUN intervention was tested in a randomized controlled trial with pre-test post-test design with five intervention and five control schools in Belgium and included children of the 5^th^ and 6^th^ grade. The children wore accelerometers for seven days at pre- and post-test. Analyses included children with valid accelerometer data for at least two weekdays with minimum 10h-wearing time and one weekend day with 8h-wearing time.

**Result:**

Final analyses included 372 children (60% girls, mean age = 10.9 ± 0.7 years). There were no significant differences in the change in sedentary time or light PA between intervention and control schools for the total sample or for the subgroup analyses by gender. However, children (specifically girls) in the intervention group had a higher decrease in moderate-to-vigorous PA than children in the control group. In the intervention group, children who lived with both parents and children with one or more siblings were less likely to reduce sedentary time after exposure to the intervention. Older children, girls and children who lived with both parents were less likely to increase light PA after the intervention.

**Conclusion:**

The UP4FUN intervention did not result in an effect on children’s sedentary time. Based on the high amounts of accelerometer-derived sedentary time in this age group, more efforts are needed to develop strategies to reduce children’s sedentary time.

## Background

The potential role of sedentary time in the development of chronic diseases and overweight and obesity in children, independent from physical activity (PA) levels, has received increased attention. A recent meta-analysis by Tremblay et al. [1] clearly showed that an increase in sedentary time was associated with negative health outcomes in 5- to 17-year-old boys and girls. Although more prospective studies of high quality are needed to confirm this relationship [2], reducing sedentary time in children has been proposed as an important aim for health promotion intervention programmes in children [3]. A recent systematic review by Leung et al. [4] revealed three studies aiming specifically at reducing sedentary time in school-aged children. The study of Robinson et al. [5], including a 18-lesson classroom curriculum to reduce TV, video and video game use, showed an improvement in anthropometric measurements related to childhood obesity and a reduction of TV and video games use. Another study of Robinson and Borzekowski [6] with an updated protocol of the previous intervention, showed a decrease in screen-time among children. The intervention study of Escobar-Chaves et al. [7] included a 2-h workshop on convenient locations (e.g. clinic, library, school) and 6 bimonthly newsletters. The researchers only found a trend towards reducing media consumption. These three intervention studies only focused on reducing screen-time and did not take other sedentary behaviours into account. Thus, it is possible that the time spent in screen-time behaviour could have been allocated to other sedentary activities [3,8,9], resulting in an equal amount of total sedentary time. It is therefore recommended to target all forms of sedentary behaviour (i.e. both screen-based activities and non-screen sedentary activities such as sitting at school and passive transportation) in an intervention programme instead of focusing on one or two specific sedentary activities [3].

The ENERGY-project (“EuropeaN Energy balance Research to prevent excessive weight Gain among Youth”-project) [10] has developed a theory-, practice- and evidence-based school intervention programme with parental involvement to reduce sedentary time among 10- to 12-year-old schoolchildren. The main purpose of the ENERGY-project intervention was to reduce and to break up children’s sedentary time at home and at school. School settings are often used for implementing health promoting interventions because of the accessibility of a large population across ethnic and socio-economic groups [11]. Parental involvement in intervention programmes is recommended, since it has been demonstrated that including the family could increase the effectiveness of school-based intervention programmes [12].

The present study aimed to examine the effect of the UP4FUN pilot intervention on 10- to 12-year-old children’s total sedentary time in Belgium. Moreover, as it could be that children spent, for example, more time in light intensity physical activities as an alternative for sedentary time [13], the second aim of this study was to examine if the intervention had an effect on children’s PA level. Finally, the third aim was to investigate demographic differences (i.e. age, gender, ethnicity, living status and having siblings) between children in the intervention group who reduced sedentary time and increased light PA (LPA) and moderate-to-vigorous PA (MVPA) at post-test from children in the intervention group who showed unfavourable changes in these behaviours. By investigating these differences, certain risk groups that are less likely to improve their behaviour after an intervention can be identified.

## Methods

### Study protocol

The pilot intervention as part of the ENERGY-project was tested in five European countries (Belgium, Germany, Greece, Hungary and Norway) in a randomized controlled trial with a pre-test post-test design including an intervention and control condition. The aim was to reach a representative sample of at least 500 children per country (250 children from minimum 5 intervention schools, 250 children from minimum 5 control schools). Accelerometer-data were collected in a subsample of 20 % in each country, except for Belgium where all children were invited to wear an accelerometer. To have the largest possible sample, only the Belgian data was used in this study.

A convenience sample of twelve primary schools was selected in Flanders, the northern part of Belgium. The principals were sent a recruitment letter and afterwards contacted by phone. Ten schools agreed to participate and all children of the 5^th^ and 6^th^ grade (including the majority of pupils born in years 1999 and 2000) in these schools were invited to participate in the intervention study. Afterwards, schools were paired according to size, gender and school socio-economic status and one school in each pair was randomly drawn to the intervention condition by the international ENERGY-project coordinator, while the remaining school was allocated to the control condition, who were offered the intervention package after the post-evaluation.

Study outcomes were assessed at baseline (prior to the intervention) and after the intervention. The intervention took place in the intervention schools and lasted for six weeks. Control schools were asked to continue the usual curriculum. Pre-test data collection occurred on schooldays in September and the beginning of October 2011, while post-data collection occurred from the end of November 2011 until the end of January 2012. Children with written consent to participate, completed a questionnaire on sedentary behaviours and related factors. One of their parents was also asked to fill in a questionnaire. Finally, all children were asked to wear an accelerometer for one week to obtain objective data on sedentary time and PA. The post-test included the same child and parent questionnaire as at the pre-test (with some extra process evaluation questions for the intervention condition) and accelerometer-data were collected for the second time. The study is registered in International Standard Randomized Controlled Trial Number Register (registration number: ISRCTN34562078). The Belgian study protocol was approved by the ethical committee of the Ghent University Hospital.

### The UP4FUN intervention

The development of the intervention was based on the five steps of the Model of Planned Promotion for Population Health [14] and the most important information is summarized in Table 1. The intervention was framed in a social ecological perspective [10,15] because of the significant influence of the family environment and individual factors. A thorough description of the development and content of the intervention will soon be written in a manuscript. The intervention within the ENERGY-project was named ‘UP4FUN’, referring to standing ‘up’ and finding ‘fun’ alternatives for sedentary activities. The six weeks lasting intervention was conducted by the teachers and included one or two lessons per week. A researcher gave a one-hour teacher training per school to all teachers conducting the intervention. Teachers were also given a teacher manual including the outline of each lesson. The materials to be handed out to the pupils were provided to the teachers at the start of the intervention. Every intervention week covered one specific theme: (1) introduction of the project, (2) awareness of sitting time, (3) evaluation of sitting time, (4) influencing factors at home, (5) possibilities for activity breaks and active transportation, and (6) Family Fun Event. The description of the intervention per week/theme is described in Table 2 with a link to the determinants and behaviour change techniques [16]. Per week/theme, the teacher handed out a newsletter (NEWS) to the children to be given to their parents. The NEWS contained of personalized messages of the children and homework tasks to be completed at home. The primary goal of the newsletters was to involve the parents in the intervention. Motivating factors were included to support the ‘fun’ aspect of the intervention (e.g. step counters and stickers) and to support public commitment to the project message (UP4FUN bracelets).

**Table 1:** Development of the intervention based on the Model of Planned Promotion for Population Health

	
**Step 1: Analysis of health and quality of health**	*Development of chronic diseases and overweight and obesity in children*
**Step 2: Analysis of risk factors**	*Role of sedentary behaviour, independent from PA*
	*Sedentary behaviour = both screen-time and non-screen time activities*
**Step 3: Analysis of determinants of risk behaviours**	*Literature reviews*
	*- importance of family environment*
	*- importance of individual factors*
**Step 4: Intervention program development**	*Stakeholders interviews on parental involvement and possible intervention activities*
	*Choosing behaviour change techniques for the identified determinants*
	*Pretesting of core components in five European countries*
**Step 5: Implementation of the UP4FUN intervention**	*Teacher training*
	*Teacher manual*

**Table 2:** Description of the UP4FUN intervention linked to the behavior change techniques

** Themes**	**Intervention components**	**Link with determinants and behavior change techniques**
*Week 1: Introduction to the project*	- Teacher gives information on the project	- Knowledge: information about the health-behaviour link (I)
	- Children start wearing bracelet	- Attitude: Intention information (I)
	- NEWS 1: ‘Welcome!’	- Knowledge: Information about health-behaviour link (IP)
*Week 2: Awareness of children’s sitting time*	- Children register sitting time for several sedentary activities (e.g. reading, TV,.)	- Awareness: self-monitoring with normative feedback (I)
	- Children register steps for three activities at home	
	- Children make a list of 3 indoor and 3 outdoor fun non-sedentary activities and share it with family	
	- NEWS 2: ‘Awareness of time spent sitting’	
*Week 3: Evaluation of the sitting time*	- Based on sitting time in week 2, children write personal goal and try it out for one week	- Awareness: Goal setting with self-rewarding use (I)
	- Children evaluate personal goal with ‘smileys’ and ‘frownies ‘(stickers)	- Automaticity: Use of prompts (I)
	- Children write down 3 difficulties regarding achieving their personal goal and 3 solutions	- Self-efficacy: Barrier identification (I)
	- NEWS 3: ‘Helping and supporting your child to aim for less sitting time’	- Awareness of child behaviour: Monitoring child behaviour (IP)
*Week 4: Influencing factors at home*	- Children write down number of pupils in class with rules about screen-time and some examples of the rules, share this with parents and discuss family rules	- Parenting rules: Opportunities for social comparison (IP)
	- Children and then parents guess number of screens at home by category (TV, computer, games) before children count them	- Availability of screens: Barrier identification (IP)
	- NEWS 4: ‘Do screens control your family life?’	
*Week 5: Breaking up sitting time and active transportation*	- Children brainstorm ideas for non-sedentary recess activities and write it down on a poster	- Role modeling: modeling and demonstrating behaviour (O)
	- Teacher conduct activity breaks per sitting lesson	- Role modeling: identification as role models (I)
	- Children are motivated to try the activity breaks at home	
	- Children are encouraged to practice active transport to school	
	- NEWS 5: ‘Short activity breaks are better than no breaks at all’	
*Week 6: Family Fun Event*	- The family participates in the Family Fun Event (to summarize the project and share experiences)	- Social support: Plan social support and social change (IP)
	- NEWS 6: ‘Thank you for taking part in the UP4FUN project’	

I, individual level; IP, interpersonal level (mainly parents); O, organizational level (teachers).

### Sedentary time and PA measurement

#### Instrumentation

Since objective measurements provide a better estimate of children’s total sedentary time and PA level [17], the present study relied on accelerometer data for measurement of sedentary time and PA. The short period to measure a large sample of children necessitated the use of all available accelerometer models, so sedentary time and PA were assessed using four models of Actigraph accelerometers (Pensacola, FL), namely the GT1M (3.8cm x 3.7cm x 1.8cm; 27g), GT3X (3.8cm x 3.7cm x 1.8cm, 27g), GT3X + (4.6cm x 3.3cm x 1.5cm, 19g) and Actitrainer (8.6cm x 3.3cm x 1.5cm, 51g). All accelerometers were worn on the right hip, secured by an elastic waist belt. We only made use of the vertical axis output of every accelerometer model for the present study. The Actitrainer, GT3X and GT3X + have identical triaxial accelerometers. Furthermore, a recent study confirmed that the vertical axis output for the GT3X is similar as for the GT1M [18].

#### Measurement protocol

Accelerometers were initialized using ActiLife software [19], selecting a 15-s epoch measurement interval. Children wore the accelerometer for seven consecutive days, including two weekend days. They were instructed to wear the accelerometer for all waking hours, but to remove it during bathing and other aquatic activities. The software Meter plus 4.2 was used to screen, score and clean the accelerometer data files of the seven days measurement [20]. Non-wearing time was calculated as periods of more than 20 min of consecutive zero counts. Children were included in the study if they had at least 2 weekdays with minimum 10h-wearing time and 1 weekend day with minimum 8h-wearing time [21]. The average counts per 15 s provided information on the overall activity level. Minutes per day of sedentary time, LPA, moderate PA and vigorous PA were estimated using the cut-points from Treuth et al. [22]: ≤100 counts per minute (cpm) equals sedentary time, 101–2999 cpm equals LPA, 3000–5199 cpm equals moderate PA and ≥5200 cpm equals vigorous PA. Moderate and vigorous PA were combined into MVPA.

### Demographic variables

Participants’ age and gender were assessed in the child’s questionnaire with single questions. Language spoken at home (native or non-native) was used as an indicator of ethnicity. The questionnaire also contained a question on the living status of children (“Which adults do you live with?”) and on having siblings.

### Statistical analyses

SPSS 15.0 (SPSS Inc, Chicago, IL) was used to describe sample characteristics. All outcomes were separately calculated for weekdays and weekend days, and during school hours and after school hours. To take into account that some children had more week- and weekend days than others, total sedentary time, LPA and MVPA per day were calculated using the following formula: ( (outcome on a weekday * 5) + (outcome on a weekend day * 2) ) / 7. All outcomes were divided by total wearing time, and were expressed in percentage of the total wearing time. We also investigated the intervention effect on the average length of the sedentary bouts (expressed in minutes). To be defined as a sedentary bout, there had to be ≥ 10 min below 100 cpm (sedentary cut-point). It was assumed that if children had a lower average length of sedentary bouts, they had interrupted their sitting time more often. Total sedentary time and number of sedentary bouts were included as a covariate. All variables were first checked for normal distribution (skewness > 0.7). The MVPA variables at pre- and post-test and the average length of sedentary bouts at pre-test had a skewness of more than 0.7, so values higher than the 90th percentile were replaced by the value that equalizes the 90th percentile to obtain a normal distribution. To study the change in sedentary time and PA according to the condition, multilevel repeated measures analysis was performed using MLwiN 2.22 (Centre for Multilevel Modelling, University of Bristol, UK) for the total sample and for boys and girls separately. Multilevel modelling (three-level: measurement-pupil-class) was used to take clustering of two measurements of children in classes into account. Age and gender were included as a covariate. Two ß-values will be reported in the results: (a) the ß-value for ‘time’ can be interpreted as the amount of change in the outcome associated with going from time 1 (pre-test) to time 2 (post-test), and (b) the ß-value for the interaction effect between ‘time’ and ‘condition’ can be interpreted as the difference in the change in the outcome going from time 1 (pre-test) to time 2 (post-test) according to the condition to which children belong (intervention vs. control condition).

To consider the effect size of significant time or interaction effects, we have reported Cohen’s d statistic (small = 0.20, moderate = 0.50, large = 0.80) [23]. Values were only reported in the text, not in the tables.

Two-level logistic regression analyses (class-pupil) were conducted in MLwiN 2.22 as well, aiming to detect differences between the group who spent less time sedentary and more time in LPA and MVPA and the group who spent more time sedentary and less time in LPA and MVPA (dummy variable). The studied variables were age (youngest/oldest group: dummy variable), gender (boys/girls: dummy variable), ethnicity (Dutch/no Dutch language spoken at home: dummy variable), living with both parents (yes/no: dummy variable), and having siblings (yes/no: dummy variable). Only children of the intervention school were included. For each independent variable, odds ratios and confidence interval were calculated. Attrition analyses comparing the children who had valid data with the children who did not have valid data were performed as a two-level logistic regression analysis (class-pupil). For all analyses, statistical significance level was set at p < 0.05.

## Results

### Sample characteristics

In total, 740 of the 772 children in ten schools had informed consent from the parents to participate in the pre- and post-test data collection (96%). At the pre-test, 566 children provided valid accelerometer data (76%). Among the omitted children, 68 did not have sufficient weekend days and 106 did not achieve sufficient week- and weekend days. At the post-test, 479 children provided valid accelerometer-data (65%). Among the omitted children, 3 did not have sufficient weekdays, 65 did not have sufficient weekend days and 192 failed in achieving sufficient week- and weekend days. In total, 386 children (52%) had valid data on the two time points (pre- and post-test). Fourteen of them had missing data on one or more demographical questions, which resulted in a final sample of 372 children (225 girls, 147 boys) with a mean age of 10.9 ± 0.7 years. There were 231 children from control schools (141 girls, 90 boys) and 141 from intervention schools (84 girls, 57 boys). Attrition analyses, comparing the 372 children with complete data to those with incomplete data (n = 368), showed no significant differences in age, ethnicity, parental living status and having siblings, but boys were twice more likely to have incomplete data than girls (OR = 1.99; 95% CI = 1.49 – 2.69).

### Effect of the UP4FUN-intervention on sedentary time, LPA and MVPA

Analyses showed no significant differences in the change in sedentary time or LPA for the total sample (Table 3), and for boys and girls separately (Tables 4, 5). However, for the total sample, significant differences were found in the change in MVPA per day (ß = −0.44, SE = 0.19, d = 0.12), on a weekday (ß = −0.58, SE = 0.20, d = 0.15), and during school hours (ß = −0.80, SE = 0.25, d = 0.17), depending on the condition. For girls (Table 4), analyses revealed significant differences in the change in MVPA on a weekday (ß = −0.59, SE = 0.25, d = 0.12), and during school hours (ß = −1.06, SE = 0.31, d = 0.18), depending on the condition. For boys (Table 5), no significant interaction effects were found.

**Table 3:** Time and interaction effects for sedentary time, LPA, MVPA in the total sample (adjusted for age and gender)

** n = 372**	**n(I) = 141**	**PRE**	**POST**	**Time**	Time * COND
	**n(C) = 231**			**ß (SE)**	**ß (SE)**
SED day	I	63.3%	66.5%	2.18 (0.59)***	0.96 (0.86)
	C	63.8%	66.0%		
SED weekday	I	64.1%	66.7%	1.07 (0.47)*	1.48 (0.78)
	C	64.8%	65.9%		
SED weekend day	I	62.2%	66.4%	4.17 (0.88)***	0.03 (1.41)
	C	63.2%	67.4%		
SED school hours	I	62.4%	62.9%	-0.22 (0.46)	0.70 (0.75)
	C	62.9%	62.7%		
SED after school	I	60.8%	65.8%	3.32 (0.67)***	1.69 (1.09)
	C	62.3%	65.7%		
SED bouts^a^	I	14.57 min	14.49 min	0.20 (0.14)	-0.28 (0.23)
	C	14.43 min	14.63 min		
LPA day	I	32.2%	30.1%	-1.51 (0.44)***	-0.56 (0.71)
	C	31.7%	30.2%		
LPA weekday	I	31.5%	28.9%	-0.89 (0.41)*	-0.83 (0.67)
	C	31.2%	30.3%		
LPA weekend day	I	34.1%	30.9%	-3.52 (0.76)***	0.29 (1.22)
	C	33.6%	30.1%		
LPA school hours	I	32.4%	32.5%	0.03 (0.40)	0.10 (0.65)
	C	32.4%	32.4%		
LPA after school	I	34.9%	31.1%	-2.30 (0.55)***	-1.48 (0.90)
	C	33.7%	31.4%		
MVPA day	I	3.9%	3.2%	-0.22 (0.12)	-0.44 (0.19)*
	C	3.6%	3.3%		
MVPA weekday	I	4.1%	3.4%	-0.12 (0.13)	-0.58 (0.20)**
	C	3.8%	3.7%		
MVPA weekend day	I	3.7%	2.7%	-0.65 (0.22)**	-0.30 (0.36)
	C	3.2%	2.6%		
MVPA school hours	I	5.0%	4.3%	0.17 (0.15)	-0.80 (0.25)**
	C	4.6%	4.7%		
MVPA after school	I	4.0%	2.9%	-1.00 (0.17)***	-0.06 (0.28)
	C	3.9%	2.8%		

^a^ adjusted for age, gender, number of sedentary bouts and total sedentary time; *SE*, standard error; COND, condition; *SED*, sedentary time; *LPA*, light physical activity; *MVPA*, moderate-to-vigorous physical activity; I, intervention group; C, control group; * p < 0.05; ** p < 0.01; *** p < 0.001.

**Table 4:** Time and interaction effects for sedentary time, LPA and MVPA in girls (adjusted for age)

** n = 225**	**n(I) = 84**	**PRE**	**POST**	**Time**	Time * COND
	**n(C) = 141**			**ß (SE)**	**ß (SE)**
SED day	I	62.7%	65.2%	1.89 (0.69)**	0.66 (1.13)
	C	64.6%	66.5%		
SED weekday	I	63.3%	65.4%	0.79 (0.60)	1.36 (0.99)
	C	65.7%	66.4%		
SED weekend day	I	62.0%	64.8%	3.32 (1.14)**	-0.53 (1.85)
	C	64.2%	67.5%		
SED school hours	I	61.8%	62.0%	-0.50 (0.58)	0.69 (0.95)
	C	63.5%	63.0%		
SED after school	I	60.4%	64.7%	3.01 (0.82)***	1.28 (1.35)
	C	63.0%	66.0%		
SED bouts^a^	I	14.43 min	14.33 min	0.22 (0.17)	-0.32 (0.28)
	C	14.48 min	14.70 min		
LPA day	I	32.6%	31.2%	-1.06 (0.56)	-0.41 (0.92)
	C	30.9%	29.9%		
LPA weekday	I	32.1%	30.7%	-0.70 (0.53)	-0.71 (0.87)
	C	30.6%	29.9%		
LPA weekend day	I	34.1%	32.1%	-2.68 (0.97)**	0.64 (1.58)
	C	32.8%	30.1%		
LPA school hours	I	32.7%	33.2%	0.15 (0.51)	0.33 (0.84)
	C	32.0%	32.2%		
LPA after school	I	35.4%	31.9%	-1.99 (0.69)**	-1.46 (1.13)
	C	33.1%	31.1%		
MVPA day	I	4.1%	3.6%	-0.15 (0.14)	-0.37 (0.23)
	C	3.4%	3.2%		
MVPA weekday	I	4.4%	3.7%	-0.03 (0.15)	-0.59 (0.25)*
	C	3.6%	3.6%		
MVPA weekend day	I	3.4%	2.9%	-0.44 (0.20)*	-0.11 (0.48)
	C	2.7%	2.3%		
MVPA school hours	I	5.3%	4.6%	0.37 (0.19)	-1.06 (0.31)***
	C	4.3%	4.7%		
MVPA after school	I	4.1%	3.2%	-1.06 (0.22)***	0.15 (0.36)
	C	3.8%	2.7%		

^a^ adjusted for age, number of sedentary bouts and total sedentary time; SE, standard error; COND, condition; SED, sedentary time; LPA, light physical activity; MVPA, moderate-to-vigorous physical activity; I, intervention group; C, control group; * p < 0.05; ** p < 0.01; *** p < 0.001.

**Table 5:** Time and interaction effects for sedentary time, LPA and MVPA in boys (adjusted for age)

** n = 372**	**n(I) = 141**	**PRE (%)**	**POST (%)**	**Time**	Time * COND
	**n(C) = 90**			**ß (SE)**	**ß (SE)**
SED day	I	63.8%	67.8%	2.69 (0.80)***	1.33 (1.28)
	C	62.4%	65.1%		
SED weekday	I	64.7%	67.8%	1.54 (0.75)*	1.61 (1.21)
	C	63.4%	65.0%		
SED weekend day	I	61.4%	67.7%	5.47 (1.35)***	0.80 (2.16)
	C	60.8%	66.3%		
SED school hours	I	62.8%	63.8%	0.22 (0.74)	0.70 (1.19)
	C	61.7%	61.9%		
SED after school	I	61.2%	67.3%	3.81 (1.13)***	2.25 (1.82)
	C	61.4%	65.2%		
SED bouts^a^	I	14.65 min	14.48 min	0.25 (0.19)	-0.42 (0.30)
	C	14.22 min	14.47 min		
LPA day	I	32.4%	29.4%	-2.26 (0.68)***	-0.73 (1.10)
	C	33.3%	31.0%		
LPA weekday	I	31.4%	29.2%	-1.24 (0.64)	-0.95 (1.03)
	C	32.5%	31.2%		
LPA weekend day	I	34.9%	29.9%	-4.86 (1.17)***	-0.14 (1.88)
	C	35.7%	30.9%		
LPA school hours	I	32.6%	32.2%	-0.16 (0.63)	-0.25 (1.02)
	C	33.5%	33.3%		
LPA after school	I	34.4%	30.1%	-2.79 (0.91)**	-1.47 (1.47)
	C	34.5%	31.7%		
MVPA day	I	3.6%	2.7%	-0.33 (0.20)	-0.54 (0.32)
	C	3.8%	3.4%		
MVPA weekday	I	3.8%	2.9%	-0.24 (0.22)	-0.58 (0.35)
	C	4.0%	3.7%		
MVPA weekend day	I	3.2%	2.2%	-0.53 (0.25)*	-0.46 (0.40)
	C	3.2%	2.7%		
MVPA school hours	I	4.4%	3.9%	-0.13 (0.25)	-0.43 (0.40)
	C	4.7%	4.6%		
MVPA after school	I	3.8%	2.4%	-1.02 (0.28)***	-0.37 (0.45)
	C	3.9%	2.9%		

^a^ adjusted for age, number of sedentary bouts and total sedentary time; SE, standard error; COND, condition; SED, sedentary time; LPA, light physical activity; MVPA, moderate-to-vigorous physical activity; I, intervention group; C, control group; * p < 0.05; ** p < 0.01; *** p < 0.001.

Significant time effects were found for sedentary time and LPA per day (ß = 2.18, SE = 0.59, d = 0.20; ß = −1.51, SE = 0.44, d = 0.18), on a weekday (ß = 1.07, SE = 0.47, d = 0.12; ß = −0.89, SE = 0.41, d = 0.11), on a weekend day (ß = 4.17, SE = 0.88, d = 0.25; ß = −3.52, SE = 0.76, d = 0.25) and after school (ß = 3.32, SE = 0.67, d = 0.26; ß = −2.30, SE = 0.55, d = 0.22) for the total sample (Table 3). Regarding MVPA, significant time effects were found on a weekend day (ß = −0.65, SE = 0.22, d = 0.16) and after school hours (ß = −1.00, SE = 0.17, d = 0.31). Several significant time effects were also found for boys and girls separately (Tables 4, 5). Effect sizes for the time effects in girls ranged between 0.12 and 0.26 and in boys between 0.11 and 0.22.

### Differences within the intervention group

Table 6 present the results of the logistic regression analyses. For sedentary time, analyses showed that children who lived with both parents were less likely to reduce their sedentary time per day (OR = 0.36, 95% CI = 0.14-0.90) and after school hours (OR = 0.40, 95% CI = 0.16-1.00) than children who did not live with both parents. Children having one or more siblings were less likely to reduce their sedentary time on a weekend day (OR = 0.22, 95% CI = 0.06-0.70) than children having no siblings. For LPA, the oldest age group was less likely to increase their LPA at school (OR = 0.31, 95% CI = 0.12-0.73) than the youngest age group. Also girls were less likely to increase their LPA level at school (OR = 0.35, 95% CI = 0.15-0.78) than boys. Finally, children who lived with both parents were less likely to increase their LPA level on a weekday (OR = 0.34, 95% CI = 0.14-0.85) than children who did not live with both parents. For MVPA, no significant results were found.

**Table 6:** Differences between the group improving and the group worsening in sedentary time and PA at post-test (intervention condition)

** n = 141**		**Independent variables**				
		**Age**	**Gender**	**Ethnicity**	**Living status**	**Having siblings**
		***(ref: Oldest age group; n = 70)***	***(ref: Girls; n = 82)***	***(ref. Dutch language; n = 129)***	***(ref. Living with both parents; n = 109)***	***(ref: Having one or more siblings; n = 122)***
**Dependent variables**						
SED day	ß (SE)	-0.22 (0.42)	-0.49 (0.42)	-0.18 (0.78)	-1.02 (0.47)*	-0.84 (0.59)
*(ref: decreasing; n = 35)*	OR (CI)	0.80 (0.35-1.84)	0.62 (0.27-1.41)	0.84 (0.18-3.84)	**0.36 (0.14-0.90)**	0.43 (0.14-1.37)
SED weekday	ß (SE)	-0.41 (0.40)	-0.35 (0.40)	1.31 (1.09)	-0.76 (0.47)	-0.13 (0.61)
*(ref: decreasing; n = 40)*	OR (CI)	0.67 (0.30-1.46)	0.70 (0.32-1.55)	3.71 (0.44-31.73)	0.47 (0.19-1.17)	0.88 (0.26-2.94)
SED weekend day	ß (SE)	-0.32 (0.40)	-0.07 (0.41)	0.69 (0.88)	-0.14 (0.49)	-1.51 (0.59)*
*(ref: decreasing; n = 40)*	OR (CI)	0.72 (0.33-1.60)	0.93 (0.42-2.07)	1.99 (0.36-11.03)	0.87 (0.33-2.27)	**0.22 (0.07-0.70)**
SED school	ß (SE)	-0.59 (0.45)	-0.49 (0.39)	0.06 (0.74)	-0.68 (0.48)	-0.02 (0.59)
*(ref: decreasing; n = 68)*	OR (CI)	0.55 (0.23-1.34)	-0.49 (0.39)	1.06 (0.25-4.52)	0.51 (0.20-1.29)	0.98 (0.31-3.15)
SED after school	ß (SE)	-0.014 (0.41)	-0.45 (0.42)	-0.32 (0.76)	-0.92 (0.47)*	1.19 (0.82)
*(ref: decreasing; n = 36)*	OR (CI)	0.99 (0.44-2.22)	0.64 (0.28-1.45)	0.73 (0.16-3.20)	**0.40 (0.16-1.00)**	3.23 (0.66-16.40)
SED bouts	ß (SE)	-0.44 (0.40)	0.30 (0.37)	0.27 (0.72)	-0.03 (0.44)	-0.67 (0.56)
*(ref: decreasing; n = 66)*^a^	OR (CI)	0.96 (0.44-2.10)	1.36 (0.66-2.80)	1.32 (0.32-5.34)	0.97 (0.41-2.28)	0.62 (0.32-1.20)
LPA day	ß (SE)	-0.33 (0.39)	-0.47 (0.40)	0.05 (0.77)	-0.71 (0.46)	-0.49 (0.34)
*(ref: increasing; n = 43)*	OR (CI)	0.72 (0.33-1.56)	0.63 (0.29-1.36)	1.05 (0.23-4.70)	0.49 (0.20-1.21)	0.62 (0.32-1.20)
LPA weekday	ß (SE)	-0.39 (0.42)	-0.37 (0.39)	0.77 (0.84)	-1.08 (0.47)*	-0.03 (0.60)
*(ref: increasing; n = 52)*	OR (CI)	0.68 (0.30-1.53)	0.69 (0.32-1.50)	0.16 (0.42-11.21)	**0.34 (0.14-0.85)**	0.97 (0.30-3.16)
LPA weekend day	ß (SE)	-0.50 (0.38)	0.25 (0.39)	-0.10 (0.71)	-0.54 (0.45)	-1.06 (0.58)
*(ref: increasing; n = 49)*	OR (CI)	0.60 (0.29-1.28)	1.28 (0.59-2.77)	0.91 (0.23-3.65)	0.58 (0.24-1.55)	0.35 (0.11-1.09)
LPA school	ß (SE)	-1.17 (0.44)**	-1.06 (0.41)*	-0.50 (0.74)	-0.92 (0.50)	0.20 (0.63)
*(ref: increasing; n = 77)*	OR (CI)	**0.31 (0.12-0.73)**	**0.35 (0.15-0.78)**	0.61 (0.14-2.60)	0.40 (0.15-1.06)	1.22 (0.35-4.20)
LPA after school	ß (SE)	-0.10 (0.40)	-0.55 (0.40)	-0.15 (0.74)	-0.68 (0.46)	0.36 (0.64)
*(ref: increasing; n = 40)*	OR (CI)	0.90 (0.42-1.96)	0.58 (0.26-1.26)	0.86 (0.20-3.71)	0.51 (0.21-1.25)	1.43 (0.41-5.01
MVPA day	ß (SE)	-0.01 (0.42)	0.74 (0.45)	0.08 (0.74)	-0.61 (0.47)	-0.12 (0.65)
*(ref: increasing; n = 35)*	OR (CI)	0.99 (0.44-2.25)	2.10 (0.87-5.07)	0.92 (0.22-3.95)	0.54 (0.22-1.37)	0.89 (0.25-3.19)
MVPA weekday	ß (SE)	0.09 (0.40)	0.34 (0.41)	0.68 (0.84)	-0.82 (0.45)	0.14 (0.65)
*(ref: increasing; n =39)*	OR (CI)	1.09 (0.50-2.38)	1.40 (0.63-3.14)	1.97 (0.38-10.18)	0.44 (0.18-1.07)	1.14 (0.32-4.06)
MVPA weekend day	ß (SE)	-0.04 (0.19)	-0.10 (0.18)	0.25 (0.40)	-0.19 (0.25)	0.13 (0.28)
*(ref: increasing; n = 43)*	OR (CI)	0.96 (0.67-1.38)	0.90 (0.63-1.30)	1.29 (0.59-2.82)	1.21 (0.74-5.43)	1.13 (0.65-1.97)
MVPA school	ß (SE)	0.08 (0.43)	0.56 (0.39)	0.34 (0.73)	-0.56 (0.46)	0.03 (0.60)
*(ref: increasing; n =54)*	OR (CI)	1.08 (0.47-2.52)	1.74 (0.81-3.75)	1.40 (0.33-5.91)	0.57 (0.43-1.41)	1.03 (0.32-3.31)
MVPA after school	ß (SE)	-0.52 (0.39)	-0.35 (0.39)	0.19 (0.75)	-0.44 (0.47)	1.43 (0.81)
*(ref: increasing; n = 43)*	OR (CI)	0.59 (0.28-1.27)	0.71 (0.33-1.53)	0.82 (0.19-3.55)	0.64 (0.26-1.62)	4.18 (0.86-20.26)

^a^ adjusted for number of sedentary bouts and total sedentary time; SED, sedentary time; LPA, light physical activity; MVPA, moderate-to-vigorous physical activity; * p < 0.05; ** p < 0.01.

## Discussion

The present study investigated the effect of a school-based pilot intervention to reduce sedentary time on objectively measured sedentary time, LPA and MVPA. Mean values showed that sedentary time was very high in Belgian school children as they spent more than 60% of their waking time in a sedentary way at the pre-test. At the post-test, this was more than 65%. This confirms that sedentary time is an important target behaviour in health promoting programmes and further efforts are needed to decrease the high amount of sedentary time in children.

Statistical analyses indicated several significant time effects for all outcomes, although the effect sizes were small. In general, there was an increase in sedentary time and decreases in LPA and MVPA for all children. This may best be explained by a seasonal trend and related temperatures, as the pre-test was conducted in September and October and the post-test mostly in December and January. Earlier research in the same age group has demonstrated that temperature is positively related to children’s PA level [24,25] and that the amount of rainfall is negatively associated with PA [24,25] and positively with sedentary time [24]. Based on official national weather online services ( http://www.meteo.be), the mean temperature was 16.5° during pre-test weeks and 5.6° during post-test weeks. Moreover, the mean amount of rainfall was 83.1 mm during pre-test weeks and 119.3 mm during post-test weeks. Thus, weather variations between pre- and post-test could have caused the significant time effects on sedentary time, LPA and MVPA, suggesting further research might take the weather influences into account.

Further, no intervention effects were found on the total sedentary time per day, on a weekday, on a weekend day, during school hours, after school hours and on the average length of the sedentary bouts. These results could be attributed to the rather short intervention period. Beside the fact that this was a pilot-test of the intervention, the reason to choose a short intervention period and a rather limited intervention was to make the intervention more feasible to perform, as the actual implementers of the intervention were the teachers, not the researchers, to increase sustainability. In many intervention studies, teachers are asked to implement the intervention in addition to the school curriculum and intervention programmes should therefore be attainable to conduct [26]. Also Wahi et al. [27] suggested that interventions to reduce sedentary time should be feasibly implemented in fewer sessions over a short period of time. However, a recent meta-analysis [3] demonstrated that interventions to reduce sedentary time with an intervention period of less than 4 months yielded small treatment effects. The researchers also stated that sedentary behaviours could have a strong habitual component and are therefore difficult to change, especially in a short intervention period. In sum, the optimal solution is probably to find a compromise between sufficient exposure and practicability for the teachers.

Another possible explanation for the disappointing results is the use of accelerometers to examine the intervention effects in this study. Objective measures provide a good estimate of children’s actual sitting time and are nowadays recommended to measure sedentary time [17]. However, because accelerometers do not distinguish between sitting and standing, it is difficult to capture activity breaks. We made the assumption that a shorter length of sedentary bout could reflect more activity breaks (if total sedentary time and the number of bouts are taken into account), but this is a rather arbitrary definition. All this suggests that inclinometers could be a better instrument to disclose intervention effects, and in particular to capture activity breaks.

Finally, the ENERGY-project including the UP4FUN intervention was grounded in a social ecological perspective, highlighting the importance of both individual and environmental factors [10,15]. The focus in this intervention was specifically on parents, but other influencing environmental factors might be taken into account as well when developing an intervention programme to induce an effect on children’s behaviour, particularly since we already stated that sedentary behaviour is a behaviour difficult to change. For example, it could be that involving the community and introducing changes in the physical environment of the school might increase the chance on intervention effectiveness.

It was further hypothesized that children could have replaced sedentary time by LPA, as light physical activities were proposed as an alternative for sedentary activities in the intervention. No significant effect was found, which could be expected as there was no effect on sedentary time as well. We also measured changes in MVPA to examine the possibility that children would have replaced a part of their sedentary time by more intensive activities. A few small statistically significant interaction effects were found, although it was in the advantage of the children in the control schools, who had an increase or a smaller decrease in MVPA in comparison to children of the intervention schools. These results indicate that an intervention specifically aiming at reducing sedentary time, might not have a positive impact on MVPA, confirming that sedentary time and MVPA are two independent behaviours. If researchers want to promote MVPA as well, a multi-component intervention may be needed to reach this goal.

The current study also compared the children in the intervention group who spent less time sedentary at the post-test with the children who spent equally or more time sedentary at the post-test. Overall, not many significant results were found, which means that for most outcomes no specific subgroups could be revealed. Results only showed that children who lived with both parents were less likely to reduce their sedentary time after the intervention than children who did not live with both parents. This is quite surprising as recent research in the same age group found that not living with married/cohabitating parents was associated with an increase in total screen-time over a time period of one year [28]. A possible explanation could be that although children lived with both parents, only one of the parents participated actively in the intervention, e.g. reading newsletters, going to the Family Fun Event. It is therefore of importance that both parents are engaged in the intervention. In addition, it was found that children having one or more siblings were less likely to reduce their sedentary time on a weekend day at the post-test than children having no siblings. It could be that children have difficulties in reducing their sedentary time at home, when their siblings are not motivated to reduce their sedentary time as well. This emphasizes the importance of support and modeling of the whole family [3,4]. Further, since girls and the oldest age group were less likely to increase their LPA level at school at the post-test than boys and younger children, teachers implementing the intervention might pay more attention to these subgroups at school. However, it must be kept in mind that these analyses were conducted in a rather small group with an unequal distribution between groups and the results should therefore be cautiously interpreted.

Study limitations included the convenience sample, the use of different accelerometer models and the relatively large drop-out of children due to the lack of valid accelerometer data. Important strengths were the randomized controlled trial with the pre-test post-test design including an intervention and control condition and the involvement of parents.

## Conclusions

Despite that the UP4FUN pilot intervention did not have an impact on Belgian children’s objectively measured sitting time, future efforts to reduce sedentary time are definitely needed, as it appears that the children in this study spent more than 60% of their waking time in a sedentary way.

## Competing interests

The authors declare that they have no competing interests.

## Author’s contributions

MV has conducted the analyses and drafted the paper. JB developed the concept and design of the ENERGY-project. EB, NL, FNV have been involved in the development and evaluation protocol of the UP4FUN intervention. MV, WVL, LM and IDB have been involved in the coordination and/or implementation of the UP4FUN study in Belgium. All authors read and approved the final manuscript.

## Pre-publication history

The pre-publication history for this paper can be accessed here:

http://www.biomedcentral.com/1471-2458/12/805/prepub
